# Intracellular folate concentrations in Down syndrome patients with arthritis

**DOI:** 10.1186/1546-0096-10-S1-A52

**Published:** 2012-07-13

**Authors:** Mara L Becker, Leon van Haandel, Nasreen Talib, Andrew Lasky, Mark F Hoeltzel, J Steven Leeder

**Affiliations:** 1 Children's Mercy Hospital, Kansas City, MO, USA

## Purpose

Patients with Down syndrome (DS) have an increased prevalence of arthritis compared to the general population, and often the arthritis is severe and debilitating. In light of the concern for folate deficiency in this patient population, and a clinically observed increased risk for methotrexate (MTX) toxicity, we investigated intracellular folate concentrations in DS patients on methotrexate therapy.

## Methods

This single center cross-sectional study evaluated 102 JIA patients on stable doses of MTX, including 4 DS patients, and 108 patients who were not currently on MTX, including 15 DS patients. After obtaining informed consent, blood was obtained during routine lab monitoring. Clinical data was collected by chart review. RBC folate concentrations were determined after deconjugation and analyzed via reversed phase separation and stable isotope dilution tandem mass spectrometry. Measured intracellular folate isoforms included: 5,10 methenyl tetrahydrofolate **(5,10-Methenyl THF)** which included the isoforms 5 formyl THF, 10 formyl THF, and 5,10 methenyl THF; 5 methyl tetrahydrofolate (**5-MTHF)** and the sum of the two. RBC MTX polyglutamate (MTXGlu) concentrations were measured by ion-pair separation followed by tandem mass spectrometry.

## Results

In subjects not on MTX therapy, folate concentrations (mean nmol/L (± SD)) tended to be lower in DS patients: 5-MTHF [**790.1**(± 404.2) vs. 1028.9 (± 487.6) *P*=0.08]; 5, 10-Methenyl THF [**55.4** (± 79.5) vs. 92.6 (± 106.5) *P*=0.005] and sum of the two [**845.5** (± 403.5) vs. 1121.6 (± 512.3) *P*=0.05]. In subjects receiving MTX, folate concentrations in the DS patients appeared to be higher: 5-MTHF [**1102** (± 153.1) vs. 665.9 (± 274.6) *P*=0.006]; 5,10-Methenyl THF [**58.4** (± 78.0) vs. 67.9 (± 76.9) P=0.4] and sum of the two [**1176** (± 195.2) vs. 733.8 (± 287.0) *P*=0.007]. When the sum folate concentrations were directly compared in subjects without DS, there was an expected anti-folate effect in subjects receiving MTX [**733.8** (± 287.0) vs. 1121.6 (± 512.3) *P*<0.0001]. However, there was no significant difference in sum folate concentrations despite being on MTX in the DS patients [**1176.1** (± 195.2) vs. 845.5 (± 403.5) *P*=0.09]. All DS patients on MTX were receiving biologics and had active arthritis at the time of blood draw. No DS patient received supplemental folate. Clinical characteristics were similar, except that DS patients were on lower doses of MTX [**0.26mg/kg** (± 0.08) vs. 0.51mg/kg (± 0.24) *P*=0.04], and total MTXGlu concentrations, total MTXGlu concentrations corrected for dose of MTX administered, and long chain MTXGlu_3-5_ were not statistically different in DS patients.

## Conclusion

Despite intracellular folate concentrations being lower in DS patients at baseline, there appears to be no significant folate inhibition by MTX in this population of patients. In our cohort of patients with JIA receiving MTX, those with active arthritis had higher intracellular folate concentrations, suggesting a less effective anti-folate effect from MTX. This may also explain the poor response to MTX in our small population of DS patients receiving MTX. However, the number of DS patients receiving MTX in our cohort is small and further assessment in a larger cohort will be necessary to confirm these findings.

## Disclosure

Mara L. Becker: None; Leon van Haandel: None; Nasreen Talib: None; Andrew Lasky: None; Mark F. Hoeltzel: None; J. Steven Leeder: None.

